# CCCH-Type Zinc Finger Family in Maize: Genome-Wide Identification, Classification and Expression Profiling under Abscisic Acid and Drought Treatments

**DOI:** 10.1371/journal.pone.0040120

**Published:** 2012-07-06

**Authors:** Xiaojian Peng, Yang Zhao, Jiangang Cao, Wei Zhang, Haiyang Jiang, Xiaoyu Li, Qing Ma, Suwen Zhu, Beijiu Cheng

**Affiliations:** Key Laboratory of Crop Biology of Anhui Province, Anhui Agricultural University, Hefei, China; Université Paris-Diderot, France

## Abstract

**Background:**

CCCH-type zinc finger proteins comprise a large protein family. Increasing evidence suggests that members of this family are RNA-binding proteins with regulatory functions in mRNA processing. Compared with those in animals, functions of CCCH-type zinc finger proteins involved in plant growth and development are poorly understood.

**Methodology/Principal Findings:**

Here, we performed a genome-wide survey of CCCH-type zinc finger genes in maize (*Zea mays* L.) by describing the gene structure, phylogenetic relationships and chromosomal location of each family member. Promoter sequences and expression profiles of putative stress-responsive members were also investigated. A total of 68 CCCH genes (*ZmC3H1*-*68*) were identified in maize and divided into seven groups by phylogenetic analysis. These 68 genes were found to be unevenly distributed on 10 chromosomes with 15 segmental duplication events, suggesting that segmental duplication played a major role in expansion of the maize CCCH family. The Ka/Ks ratios suggested that the duplicated genes of the CCCH family mainly experienced purifying selection with limited functional divergence after duplication events. Twelve maize CCCH genes grouped with other known stress-responsive genes from *Arabidopsis* were found to contain putative stress-responsive *cis*-elements in their promoter regions. Seven of these genes chosen for further quantitative real-time PCR analysis showed differential expression patterns among five representative maize tissues and over time in response to abscisic acid and drought treatments.

**Conclusions:**

The results presented in this study provide basic information on maize CCCH proteins and form the foundation for future functional studies of these proteins, especially for those members of which may play important roles in response to abiotic stresses.

## Introduction

Zinc finger motifs in proteins consist of cysteines and/or histidines which coordinate a zinc ion to form local peptide structures that are required for specific biological functions [1]. Based on their structural and functional diversities, zinc finger proteins have been categorized into at least 14 families, such as ERF, WRKY, DOF and RING-finger families [2], [3], [4], [5]. CCCH-type zinc finger proteins, one group of these zinc finger families, are shown to contain tandem zinc-binding motifs characterized by three cysteines followed by one histidine [6], [7]. A typical CCCH protein usually contains 1–6 CCCH-type zinc finger motifs. Based on the different numbers of amino acid spacers between cysteines and histidines in the CCCH motif, a consensus sequence for these motifs was defined as C-X_4–15_-C-X_4–6_-C-X_3_-H (X represents any amino acid) based on the whole-genome analysis of rice and *Arabidopsis* CCCH proteins [8].

CCCH-type zinc finger proteins are RNA-binding proteins by virtue of the ability of their defining motif to directly bind to RNA, whereas most of the other zinc finger families are confirmed as DNA-binding or protein-binding proteins [9]. Although increasing evidence have demonstrated that CCCH zinc finger proteins possess RNA binding activity, the precise role of this zinc finger motif is poorly understood. CCCH motif-containing proteins comprise a large protein family, and are widely distributed across eukaryotes. In animals, tristetraprolin (TTP) is a well-characterized CCCH zinc finger protein, which contains two such motifs and binds to AU-rich elements in the 3′-untranslated region of their target transcripts, in most cases mediating mRNA degradation [10], [11]. Another CCCH-type zinc finger protein, zinc-finger antiviral protein (ZAP) isolated from Rat2 fibroblasts, can directly bind to specific viral RNA sequences through its CCCH zinc finger motifs and inhibit retroviral RNA production [12]. Yet other CCCH proteins can control the translation of their target mRNAs. For example, the Drosophila protein ZC3H3 regulates mRNA nuclear adenylation and export [13].

In *Arabidopsis*, HUA1, a CCCH-type zinc finger protein with six tandem CCCH motifs, has been identified as an RNA-binding protein and likely participates in a new regulatory mechanism for flower development. This protein is able to associate with AGAMOUS mRNA so that it can indirectly determine organ identity specification [14]. AtCPSF30, the *Arabidopsis* ortholog of the 30-kD subunit of the cleavage and polyadenylation specificity factor, was shown to be a nuclear-localized RNA-binding protein that binds calmodulin [15]. In addition, some CCCH zinc finger proteins have been demonstrated to be involved in abiotic and biotic stresses. For example, salt stress-inducible zinc finger protein 1 (AtSZF1) and AtSZF2 were found to be involved in modulating the tolerance of *Arabidopsis* plants to salt stress [16]. Recently, GHZFP1, a CCCH-type zinc finger protein from cotton, was revealed to enhance tolerance to salt stress and resistance to fungal disease in transgenic tobacco [17]. Although the majority of the CCCH motifs bind RNA, DNA binding CCCH-type zinc finger proteins have also been confirmed. PEI1, an embryo-specific transcription factor in *Arabidopsis*, has been shown to play an important role during embryogenesis [18].

Although CCCH-type zinc finger proteins are known to have important roles in various aspects of plant growth and development, their functions remain poorly characterized in maize. Recently, the maize genome was completed with high quality sequences [19], which provided an opportunity to perform a genome-wide analysis of the CCCH gene family in order to understand the evolutionary history and functional mechanisms of its members in such an important species. In this study, a comprehensive analysis of the CCCH gene family was performed by searching the entire maize genome. In addition, we investigated the expression patterns of seven potential stress-responsive genes in five representative tissues and their responses to abscisic acid (ABA) and drought treatments. The results provide an important foundation for future cloning and functional studies of CCCH proteins in maize.

## Results

### Identification of CCCH Proteins in Maize

**Figure 1:**
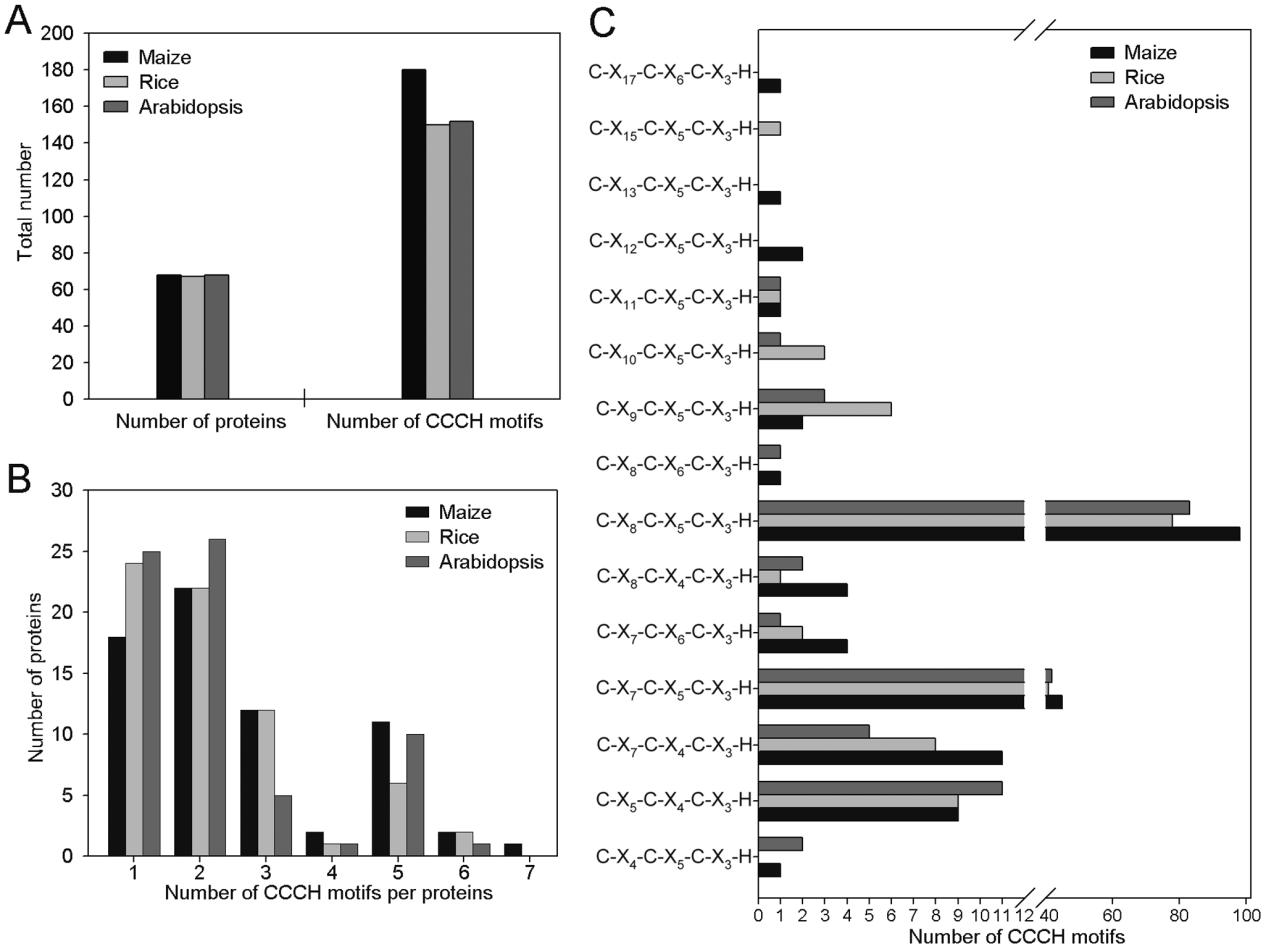
Characterization of CCCH-type zinc finger proteins. (**A**) Numbers of CCCH proteins and CCCH zinc finger motifs identified in maize, rice and *Arabidopsis*. (**B**) Numbers of CCCH proteins containing 1, 2, 3, 4, 5, 6 or 7 CCCH zinc finger motifs in maize, rice and *Arabidopsis*. (**C**) Numbers of each type of CCCH zinc finger motifs in maize, rice and *Arabidopsis*.

After extensive searches of the maize genome database by using previously reported *Arabidopsis* and rice CCCH proteins as BLASTP queries, a total of 68 CCCH genes (designated *ZmC3H1* to *ZmC3H68*) were identified in maize and listed in Table S1. Our results showed that the maize and *Arabidopsis* genomes encode the same number of CCCH proteins, which was similar to that in rice (67 members). Like *Arabidopsis* and rice CCCH proteins, the zinc finger motif in the maize CCCH zinc finger family is highly conserved. All identified CCCH genes encode proteins varying from 160 to 1200 amino acids, with a few exceptionally longer or smaller proteins (Table S1). However, some novel patterns and features of the maize CCCH zinc finger motif that differ from other plants were also found. A total of 180 CCCH zinc finger motifs were recognized by Pfam and SMART among the 68 maize CCCH proteins, which was greater than those found in *Arabidopsis* (152) and rice (150), even though similar numbers of CCCH proteins were identified in the three plants (Fig. 1A).

Previous studies have indicated that CCCH proteins have between one and six CCCH-type zinc finger motifs characterized by three cysteines followed by one histidine. However, our analysis detected a novel maize CCCH protein (*ZmC3H3*), which contains seven copies of the CCCH zinc finger motifs. Therefore, we divided the maize CCCH proteins into seven groups according to the number of copies of CCCH motif in each protein. As shown in Fig. 1B, approximately 59% of all identified maize CCCH proteins have one or two CCCH motifs, while 17.6% contain three copies and 16% have five copies. Similar trends were also found in *Arabidopsis* and rice (Fig. 1B). After whole-genome analysis of CCCH proteins in *Arabidopsis* and rice, the CCCH zinc finger proteins were re-defined as C-X_4–15_-C-X_4–6_-C-X_3_-H by Wang et al. (2008) based on the different number of amino acid spacers between cysteines and histidines, which were originally defined as C-X_6–14_-C-X_4–5_-C-X_3_-H. In this study, a novel zinc finger motif, C-X_17_-C-X_6_-C-X_3_-H, was identified in *ZmC3H17*. Similar to that reported in *Arabidopsis* and rice, the most abundant CCCH zinc finger motifs were found to be C-X_8_-C-X_5_-C-X_3_-H (54.4%) and C-X_7_-C-X_5_-C-X_3_-H (25%) in the maize CCCH family. It is worth noting that C-X_7_-C-X_4_-C-X_3_-H, C-X_5_-C-X_4_-C-X_3_-H, C-X_7_-C-X_6_-C-X_3_-H and C-X_8_-C-X_4_-C-X_3_-H are the largest groups of motifs among the 20.6% uncommon CCCH zinc finger motifs (Fig. 1C). Detailed information on the maize CCCH zinc finger family can be found in Table S2.

**Figure 2:**
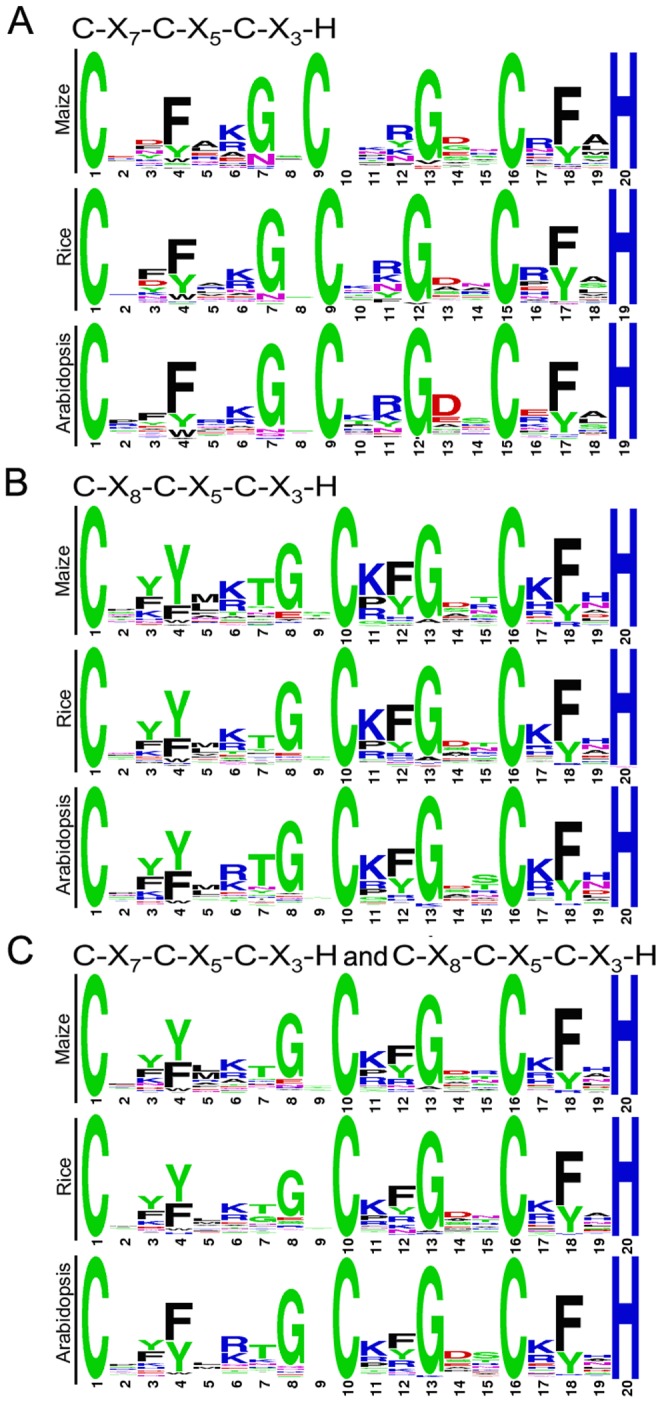
Sequence logos for common CCCH zinc finger motifs. (**A**) C-X_7_-C-X_5_-C-X_3_-H motifs of maize, rice and *Arabidopsis*. (**B**) C-X_8_-C-X_5_-C-X_3_-H motifs of maize, rice and *Arabidopsis*. (**C**) C-X_7_-C-X_5_-C-X_3_-H and C-X_8_-C-X_5_-C-X_3_-H motifs of maize, rice and *Arabidopsis*.

To explore sequence characteristics of the most common zinc finger motifs in the maize CCCH gene family, we performed sequence alignments of 45 C-X_7_-C-X_5_-C-X_3_-H motifs and 98 C-X_8_-C-X_5_-C-X_3_-H motifs to generate sequence logos, which were then compared with the corresponding CCCH motif sequence logos in rice and *Arabidopsis*. Moreover, a combined sequence logo for the two types of motifs was also created using the same method. The results confirmed that the CCCH zinc finger motif is highly conserved in the CCCH zinc finger family. As shown in Fig. 2, there was very little difference among the sequence logos derived from the three plants. Each logo of the three types of motifs was similar to the Pfam or SMART sequence logos for CCCH zinc finger motifs, especially at the four amino acids, three cysteines and one histidine, which are completely conserved among all the CCCH motifs. Differences were only found in the combined sequence logos for C-X_8_-C-X_5_-C-X_3_-H and C-X_7_-C-X_5_-C-X_3_-H motifs of the three plants. For example, tyrosine was more conserved than phenylalanine at position C_1_+3 in both of the maize and rice combined logos, while the reverse case was observed in the *Arabidopsis* combined logo (Fig. 2C).

### Phylogenetic and Structural Analysis of Maize CCCH Zinc Finger Proteins

**Figure 3:**
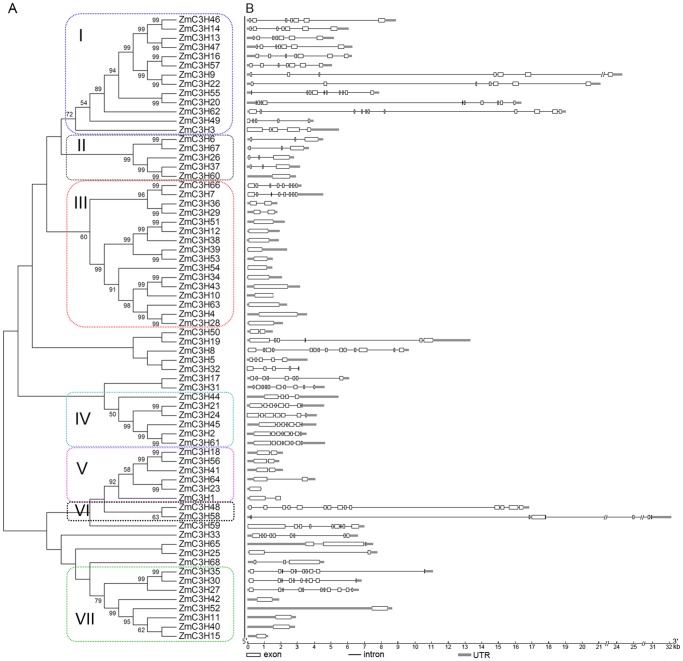
Phylogenetic relationship and exon-intron structure of maize CCCH proteins. (**A**) The unrooted tree was constructed using MEGA4.0 by the N-J method. Bootstrap values (above 50%) from 1,000 replicates are indicated at each node. (**B**) Exons and introns are indicated by white rectangles and thin lines, respectively. The untranslated regions (UTRs) are indicated by thick lines.

To detect the evolutionary relationships within the maize CCCH zinc finger family, a neighbor-joining (N-J) tree was constructed based on the alignment of the full-length sequences of the 68 maize CCCH proteins. According to the phylogenetic analysis, maize CCCH proteins were divided into seven groups (group I to group VII) (bootstrap values >50%). Sixty-eight maize CCCH genes formed 24 sister pairs, and 18 of them showed high bootstrap support (99%). We also noted that while the majority of the phylogenetic clades had well-supported bootstrap values, the phylogenetic relationships of some proteins were unclear and the bootstrap values were low at the nodes (Fig. 3A). To support the phylogenetic reconstruction, we executed an exon-intron analysis by comparing the predicted coding sequence (CDS) with the genomic sequence of the maize CCCH genes (Fig. 3B). Consistent with the phylogenetic analysis, genes clustered in the same group displayed the similar exon-intron structures, especially in the number of introns, although exceptions to this observation were also found. For example, *ZmC3H35*, -*30* and -27 in group VII contain multiple introns, while *ZmC3H42*, -*52*, -*11*, -*40* and -*15* do not contain any introns. Moreover, the intron length is also highly variable, ranging from several tens to approximately 16,000 bases. Those observations reflect the high sequence diversity of the CCCH zinc finger family. Although the sequences between adjacent cysteines of the zinc finger motif in the CCCH gene family are highly conserved, the number of motifs encoded by each protein and spacing sequences between tandem CCCH zinc finger motifs are diverse.

**Figure 4:**
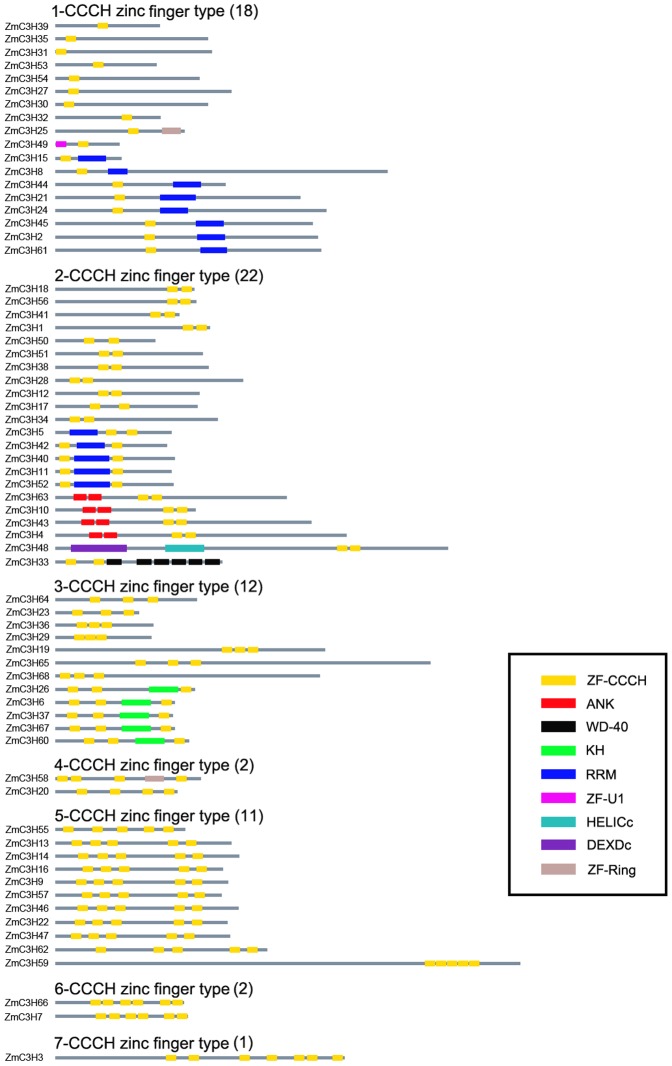
Schematic structures of maize CCCH proteins. Schematic structures of 68 CCCH zinc finger proteins identified in maize are shown with names of all the members on the left side of the figure. Different domains are indicated by different boxes denoted at the right bottom corner. Domain abbreviations: ZF-CCCH, CCCH-type zinc finger domain; ANK, ankyrin repeat domain; WD-40, WD-40 repeat domain; KH, K homology domain; RRM, RNA recognition motif; ZF-U1, U1-like zinc finger domain; HELICc, helicase superfamily C-terminal domain; DEXDc, DEAD-like helicases superfamily domain; ZF-Ring, Ring-type zinc finger domain. The proteins were grouped manually according to the number of CCCH zinc finger and the distribution of other conserved domains.

Besides containing 1–7 copies of CCCH zinc fingers, some CCCH proteins also carry several other known functional domains, including ANK, WD-40, KH, RRM, ZF-U1, HELICc, DEXDc and ZF-Ring (Fig. 4). It is worth mentioning that among the 68 members of the maize CCCH family, 13 CCCH proteins contain the RRM domain, and 5 members contain the KH domain. RRM and KH domains are common RNA-binding domains in eukaryotes [20], [21]. Moreover, RRM and KH domain-containing proteins have been demonstrated to play essential roles in many aspects of RNA metabolism, suggesting that the 18 CCCH proteins harboring these domains may function as RNA-binding proteins and are involved in RNA processing. We also noted that the majority of the CCCH proteins in the same phylogenetic subfamily displayed a similar domain architecture. For example, all of the group I proteins contain five copies of the CCCH zinc finger domain except for *ZmC3H20*, -*49* and -*3*. In particular, subfamily-specific domains were also found among the CCCH proteins. For example, the group II CCCH proteins, including *ZmC3H6*, -*67*, -*26*, -*37* and -*60*, have a conserved KH domain, suggesting that the KH domain may play important subfamily-specific functions. Thus, the phylogenetic reconstruction was further supported by analysis of the domain architecture.

**Figure 5:**
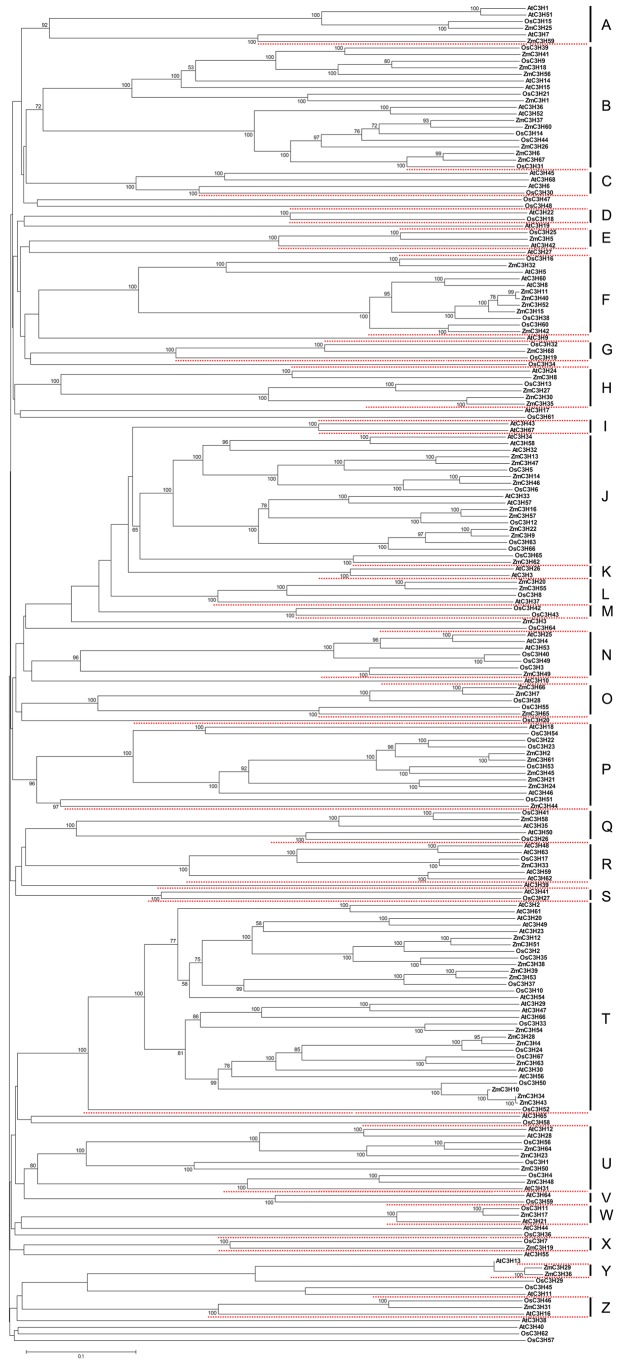
Phylogenetic relationships of maize, rice and *Arabidopsis* CCCH proteins. The tree was constructed using the ClustalX program by the N-J method from alignment of the full-length amino acid sequences of maize, rice and *Arabidopsis* CCCH proteins. Bootstrap values of 1,000 replications were executed, and only results above 50% are shown at each node.

With the development of comparative genomics, it is possible to analyze the same protein families among different species. To evaluate the evolutionary relationships of CCCH genes in maize, rice and *Arabidopsis*, a combined N-J tree was constructed from alignments of the complete sequences of all 203 CCCH proteins from the three species. We divided the 203 members into 26 groups, designated from A to Z, according to the phylogenetic clades with at least 50% bootstrap support. The phylogenetic analysis showed that most maize CCCH genes clustered with their rice or *Arabidopsis* counterparts with high bootstrap support (Fig. 5). Furthermore, we noted that *ZmC3Hs* were more closely grouped with *OsC3Hs* than with *AtC3Hs*. For instance, ortholog pairs of maize and rice CCCH proteins were more prevalent in the tree, which indicated that some ancestor CCCH genes have been existed before the divergence of maize and rice. The phylogenetic analysis confirmed the close relationship of maize and rice, consistent with the evolutionary relationships among the three species. Generally speaking, the monocot CCCH genes formed sister pairs with the nearest monocot orthologs, while the dicot CCCH genes formed sister pairs with the nearest dicot orthologs. However, sister pairs formed by *Arabidopsis* and maize/rice orthologs with very strong bootstrap support were also found in our phylogenetic tree, such as *AtC3H7*-*ZmC3H59* and *AtC3H22*-*OsC3H18*, Notably, while some groups in the phylogenetic tree consisted of a few CCCH genes of maize, rice and *Arabidopsis*, several groups were significantly expanded. For example, group T contained 32 CCCH members with well-supported bootstrap values. Generally, genes clustered into the same group of phylogenetic tree often share similar functional features. A previous study showed that the *Arabidopsis* CCCH genes clustered in the group T in our phylogenetic tree are involved in responses to abiotic stress, suggesting that maize CCCH genes of this group may play essential roles in plant stress responses [8]. CCCH genes with unclear phylogenetic relationships were also found in the combined tree (Fig. 5).

### Chromosomal Locations and Gene Duplication

**Figure 6:**
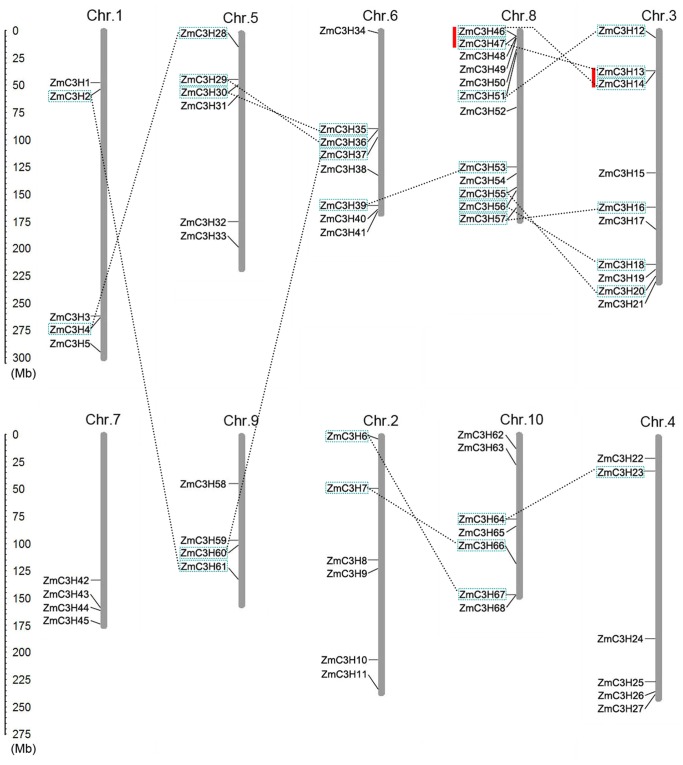
Locations of 68 CCCH proteins on 10 maize chromosomes. The scale on the left is in megabases. Chromosome numbers are indicated at the top of each bar. The gene names on the left side of each chromosome correspond to the approximate locations of each CCCH gene. The chromosome order was arranged to bring duplicated regions in close proximity. The segmental duplication genes are connected by dashed lines, and the tandem duplicated gene clusters are marked by red bars.

Based on the starting position of each gene on the chromosomes, the 68 maize CCCH genes were found to be unevenly distributed on chromosomes 1 to 10 (Fig. 6). Chromosome 8 contained the largest number of maize CCCH genes (12) followed by chromosome 3 (10). By contrast, chromosomes 7 and 9 contained the least number of maize CCCH genes (4). Eight CCCH genes were located on chromosome 6, six on chromosomes 5, 2 and 4, seven on chromosome 10 and five on chromosome 1.

Based on the phylogenetic analysis and the chromosomal locations of the *ZmC3H* genes, 15 of the 24 sister pairs were located on the same duplicated chromosomal blocks as previously reported [22]. On the other hand, a total of 30 maize CCCH genes were involved in the segmental duplications (Fig. 6). The highest frequency of segmental duplication events occurred between chromosomes 8 and 3, which showed six segmental duplication events, while no duplication events occurred among the four CCCH genes on chromosome 7. In addition, two gene clusters (*ZmC3H13/14* and *ZmC3H46*/*47*) were detected on chromosomes 8 and 3. Gene clusters may develop as a result of the birth and death process of tandem duplication, indicating that genes in the two clusters were involved in tandem duplication, consistent with the definition of tandem duplication detection in this study. Interestingly, the four tandem duplicated genes were also mapped on the same duplicated chromosomal blocks.

To explore different selective constrains on duplicated CCCH genes, the Ks and Ka/Ks ratio for each pair of duplicated CCCH genes were calculated. Generally, a Ka/Ks ratio >1 indicates accelerated evolution with positive selection, a ratio  = 1 indicates neutral selection, while a ration <1 indicates negative or purifying selection. In this study, all of the 17 duplicated pairs in the maize CCCH family were investigated. The results showed that the Ka/Ks ratios for 15 duplicated pairs were <1, with most of them being even less than 0.4, suggesting strong purifying selection (Table 1). However, the other two duplicated pairs seemed to be under positive selection, as their Ka/Ks ratios were >1. These results suggested that functions of the duplicated genes did not diverge much along with the genome evolution after the duplication events. Based on the Ka/Ks analyses, we concluded that purifying selection may be largely responsible for maintenance of function in the maize CCCH family. We also calculated the duplication dates by calculating the synonymous substitutions between the 17 duplicated CCCH gene pairs. On the basis of a substitution rate of 6.5×10^−9^ substitutions per site per year [23], [24], the duplication events for the 15 segmental duplications were estimated to have occurred approximately between 5 to 28 million years ago (Mya). However, the duplication times for the two tandem pairs of *ZmC3H13*/*14* and *ZmC3H46*/*47* were 62.92 and 74.23 Mya, respectively (Table 1). The results suggested that the two tandem duplication events occurred before the formation of the segmental duplication pairs *ZmC3H46*/*14* and *ZmC3H47*/*13*.

**Table 1:** Ka/Ks analysis and estimated divergence time for the duplicated *ZmC3H* paralogs.

Duplicated pairs	Ka	Ks	Ka/Ks	Purifying selection	Date (Mya)	Duplicate type
ZmC3H14-ZmC3H46	0.044	0.143	0.308	Yes	11	Segmental
ZmC3H13-ZmC3H47	0.063	0.168	0.375	Yes	12.92	Segmental
ZmC3H16-ZmC3H57	0.04	0.201	0.199	Yes	15.46	Segmental
ZmC3H20-ZmC3H55	0.205	0.244	0.84	Yes	18.77	Segmental
ZmC3H6-ZmC3H67	0.066	0.24	0.275	Yes	18.46	Segmental
ZmC3H37-ZmC3H60	0.134	0.131	1.023	No	10.08	Segmental
ZmC3H7-ZmC3H66	0.044	0.175	0.251	Yes	13.46	Segmental
ZmC3H29-ZmC3H36	0.014	0.169	0.083	Yes	13	Segmental
ZmC3H12-ZmC3H51	0.059	0.172	0.343	Yes	13.23	Segmental
ZmC3H39-ZmC3H53	0.061	0.178	0.343	Yes	13.69	Segmental
ZmC3H4-ZmC3H28	0.025	0.23	0.109	Yes	17.69	Segmental
ZmC3H2-ZmC3H61	0.031	0.205	0.151	Yes	15.77	Segmental
ZmC3H18-ZmC3H56	0.095	0.067	1.418	No	5.15	Segmental
ZmC3H23-ZmC3H64	0.067	0.359	0.187	Yes	27.62	Segmental
ZmC3H30-ZmC3H35	0.053	0.156	0.34	Yes	12	Segmental
ZmC3H13-ZmC3H14	0.262	0.818	0.32	Yes	62.92	Tandem
ZmC3H46-ZmC3H47	0.292	0.965	0.303	Yes	74.23	Tandem

### Analysis of the Promoters and Sequence Characteristics of Potential Abiotic Stress-responsive CCCH Genes

**Figure 7:**
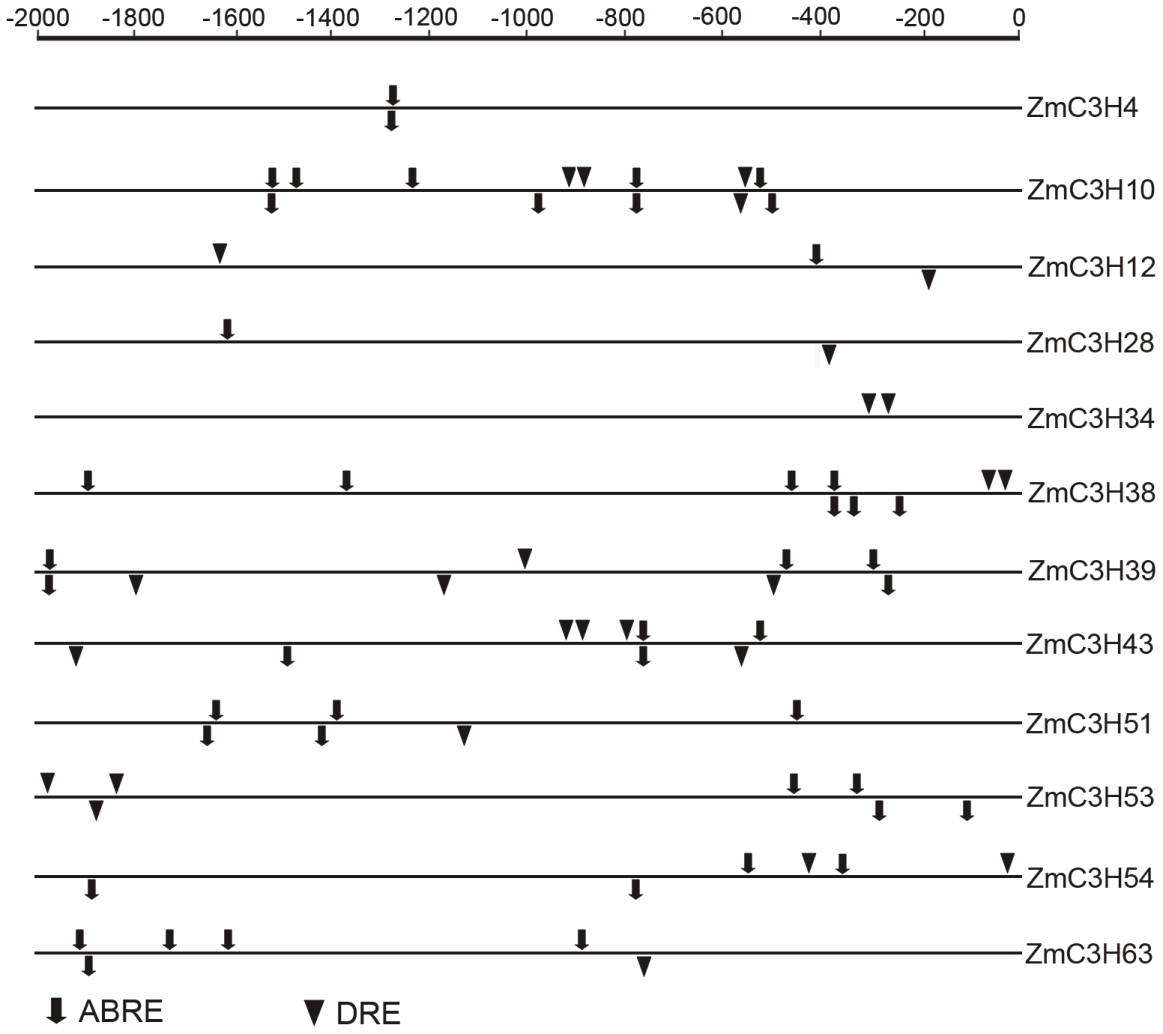
*Cis*-elements in the promoter regions of stress-responsive CCCH genes. The stress-responsive *cis*-elements distributed on the sense strand and reverse strand are shown above and below the black lines, respectively. ABRE and DRE core sequences are indicated by drop-down arrows and triangles, respectively.

As mentioned above, the *Arabidopsis* genes clustered in the group T were shown to be involved in response to abiotic stress. The phylogenetic analysis indicated that group T contains 12 maize CCCH genes and that are closely related to the *Arabidopsis* stress-responsive genes (Fig. 5). This observation prompted us to investigate possible stress-responsive *cis*-elements in the promoter regions of the 12 maize CCCH genes by searching against the PLACE database. Two types of *cis*-elements, including the ABA responsive element (ABRE) and dehydration-responsive element (DRE), were detected in the current study [25], [26]. The results showed that each of the 12 CCCH genes contain ABRE or DRE in their 2,000 bp promoter sequences, which may be responsive for their stress responsiveness (Fig. 7). Although the phylogenetic analysis showed close relationships of the 12 stress-responsive genes, we found surprising differences in the numbers of the two *cis*-elements in their promoter regions. For example, the promoter regions of *ZmC3H10* and -*43* contain multiple putative ABREs and DREs. In contrast, only two DREs were detected in the promoter of *ZmC3H34*. Moreover, we also found that the two *cis*-elements are not conserved in the promoter regions of the three sister pairs involved in segmental duplication (*ZmC3H12*/*51*, *ZmC3H39*/*53* and *ZmC3H4*/*28*), consistent with the previous finding that *cis*-elements of segmental duplication genes in *Arabidopsis* are less conserved when compared to tandem duplication genes [27]. Although this observation indicated that the three segmental duplication pairs of maize CCCH family might not have some similar regulatory features, each of the segmental duplication genes contain at least one ABRE element in their promoter regions, which is a central *cis*-element of ABA signal transduction in response to stress treatments [28]. Thus, we concluded that the duplicated genes may share similar regulatory pathway in some respects. Notably, while most of the genes contain both types of stress-responsive elements in their promoter sequences, only one type of stress-responsive element was detected in the promoter regions of *ZmC3H4* and -*34*.

**Figure 8:**
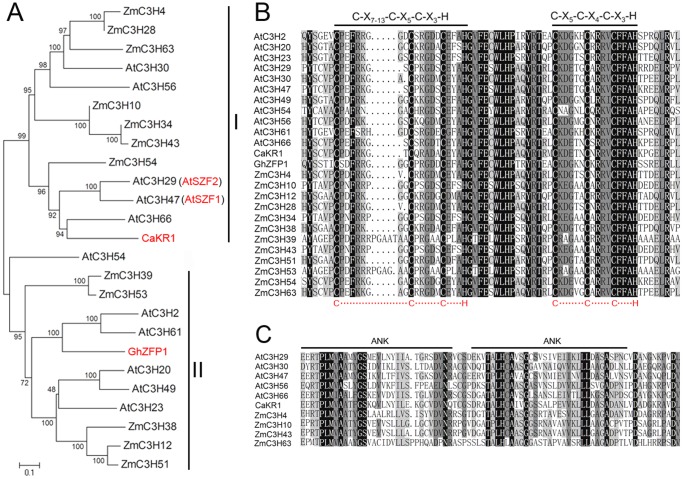
Phylogenetic relationships and sequence analysis of stress-responsive CCCH genes. (**A**) The phylogenetic tree was constructed using MEGA4.0 by the N-J method. Bootstrap values (above 50%) from 1,000 replicates are indicated at each node. (**B**) Sequence alignment of CCCH zinc finger motifs of the stress-responsive CCCH genes. The conserved CCCH zinc finger motifs are indicated by straight lines. (**C**) Sequence alignment of ANK repeat motifs of the stress-responsive CCCH genes. The conserved ANK motifs are indicated by straight lines.

To our knowledge, only four CCCH-type zinc finger proteins (CaKR1, GhZFP1, AtSZF1 and AtSZF2) that confer tolerance to stresses in transgenic plants have been isolated [16], [17], [29]. Thus, an additional N-J tree was built for the stress-responsive members using the full-length amino acid sequences. The phylogenetic tree categorized the 25 stress-responsive proteins into two distinct subfamilies (I and II) with very strong bootstrap support (Fig. 8A). Subfamily I contained 13 members, and subfamily II contained 12 members. Sequence alignments showed that each of the 25 CCCH proteins contain two putative CCCH zinc finger motifs of C-X_7–13_-C-X_5_-C-X_3_-H and C-X_5_-C-X_4_-C-X_3_-H (only one CCCH motif was detected in *ZmC3H39*, -*53* and -*54* by Pfam or SMART) (Fig. 8B). It should be noted that all of the C-X_5_-C-X_4_-C-X_3_-H motif-containing proteins of the maize and *Arabidopsis* CCCH families clustered into the same phylogenetic clade. Moreover, the C-X_5_-C-X_4_-C-X_3_-H motif contained in subfamily I proteins were found to be highly conserved, suggesting the crucial roles of this motif in subfamily-specific functions. Among the 13 CCCH genes of subfamily I, 10 genes with ankyrin (ANK) repeat motifs were identified (Fig. 8C). The ANK repeat motif has been shown to mediate protein-protein interactions, and it is found in numerous proteins with diverse functions, such as signal transduction, transcriptional control and cell cycle regulation [30], [31], [32]. These observations prompted us to further analyze the subfamily I CCCH genes.

### Expression Levels of Maize CCCH Genes in Various Tissues and in Response to Abiotic Stress

**Figure 9:**
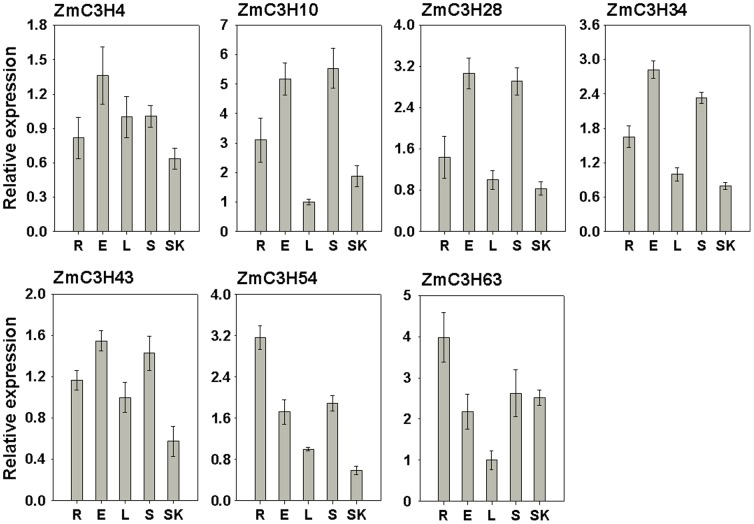
Expression of seven stress-responsive CCCH genes in five representative maize tissues. The *X*-axis is the representative tissues: R, root; E, ear; L, leaf; S, stem; and SK, silk. The *Y*-axis is the scale of relative expression level of the gene compared to the expression level in leaf. Error bars, ± SE.

Analyses of the sequences and evolutionary relationships indicated that seven maize CCCH genes were clustered in the subfamily I, including *ZmC3H4*, -*28*, -*63*, -*10*, -*34*, -*43* and -*54*, which may be a novel subfamily involved in abiotic stress responses (Fig. 8A). Thus, we used quantitative real-time PCR (qRT-PCR) to survey the relative expression levels of these seven members in five representative tissues. The results showed that the seven genes exhibit differential expression patterns in the five tissues except for *ZmC3H34* and -*43* (Fig. 9). Five of the genes, *ZmC3H4*, -*28*, -*10*, -*34* and -*43*, showed high expression levels in ears and stems, while *ZmC3H54* and -*63* were abundantly expressed in roots. In addition, the results also showed that most of the maize CCCH genes in the subfamily I exhibited lower expression levels in leaves and silks than in the other three tissues.

**Figure 10:**
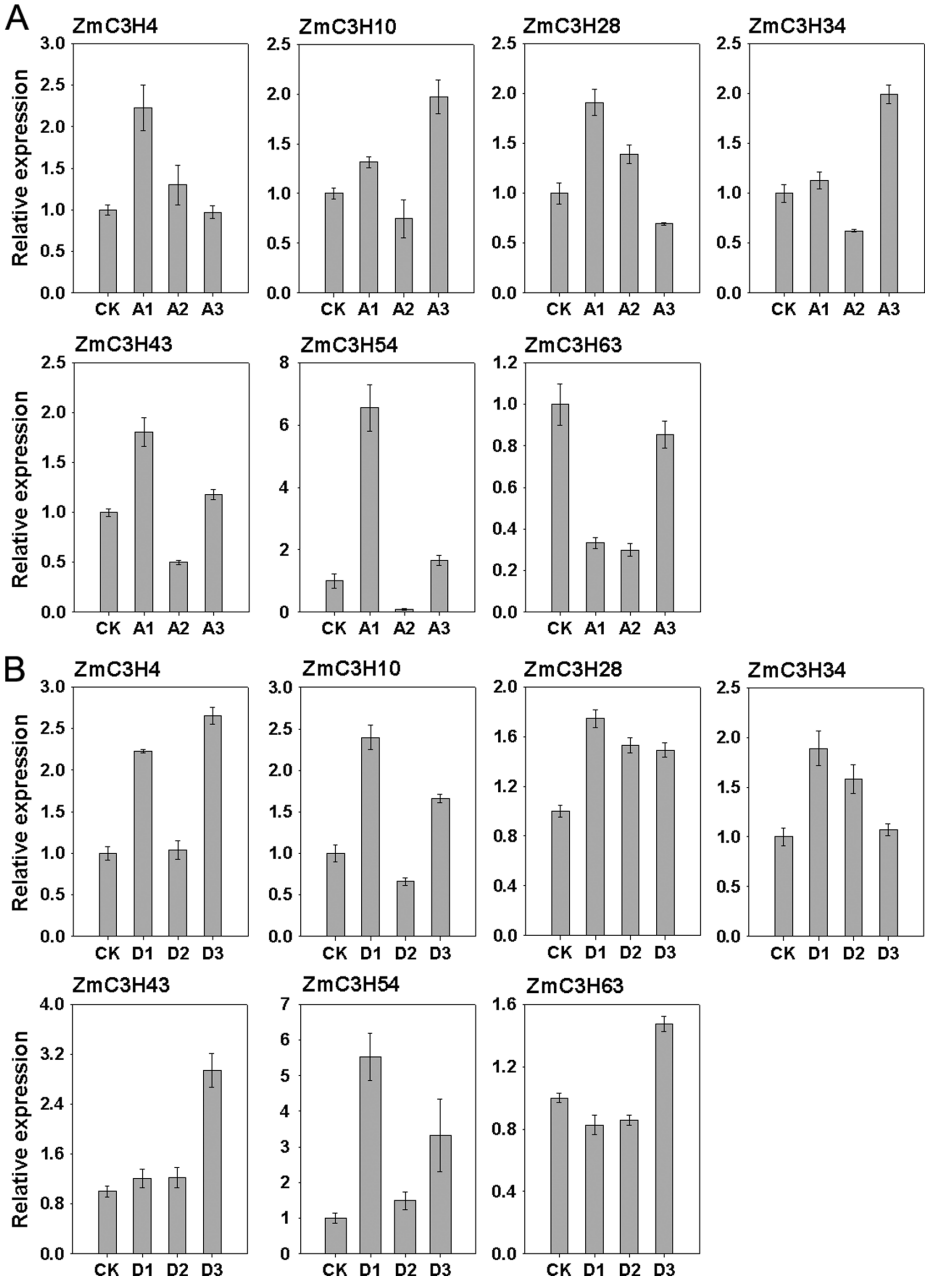
Expression of seven stress-responsive CCCH genes under stress treatments. The *Y*-axis is the scale of relative expression levels. The *X*-axis is time courses of stress treatments. Error bars, ± SE. (**A**) Realtive expression levels of the seven stress-responsive CCCH genes in response to ABA treatment. Seedlings were sampled at 0 h (CK), 3 h (A1), 6 h (A2) and 12 h (A3) after ABA treatment. (**B**) Realtive expression levels of the seven stress-responsive CCCH genes in response to drought stress. Seedlings were sampled at 0 h (CK), 3 h (D1), 6 h (D2) and 12 h (D3) after drought treatment.

Since expression patterns can provide important clues for possible gene function, we further investigated the expression levels of these genes in response to abiotic stress by subjecting three-week-old seedling leaves to ABA and drought treatments. The results showed that expression levels of all seven genes were induced or repressed by the two stress treatments, although the induction of some genes was slight (Fig. 10A and B). Under ABA treatment, *ZmC3H4*, -*28*, -*43* and -*54* were highly expressed at a relatively early stage (3 h after treatment), whereas *ZmC3H10* and -*34* levels were peaked at 12 h after treatment. *ZmC3H63* was down-regulated by ABA treatment across all time points. Notably, *ZmC3H54* was strongly up-regulated (>2-fold) at 3 h after ABA treatment but was dramatically down-regulated thereafter (Fig. 10A). Under drought stress, the highest expression levels of *ZmC3H28*, -*10*, -*34* and -*54* were found at 3 h after treatment, while those of *ZmC3H4*, -*63* and -*43* were observed later (12 h after treatment). Similar to its response to ABA treatment, *ZmC3H54* was strongly up-regulated at 3 h after drought treatment (Fig. 10B). In addition, *ZmC3H43* was also significantly up-regulated by drought stress. By comparing the expression patterns of the two segmental duplicated genes (*ZmC3H4* and -*28*) in subfamily I, we found that the two duplicated genes exhibited similar expression profiles following ABA treatment but showed little difference under drought treatment.

## Discussion

CCCH-type zinc finger proteins comprise a large superfamily and play important roles in many aspects of plant growth and development. Compared to animals, few CCCH proteins have been studied functionally in plants. In this study, we identified 68 CCCH genes in the maize genome and divided them into seven groups based on phylogenetic analysis. This study of CCCH proteins in maize confirms many patterns observed in other species, but some novel features that differ from those seen in other species were also found.

Previous studies revealed that CCCH proteins have between 1 and 6 CCCH zinc finger motifs defined as C-X_6–14_-C-X_4–5_-C-X_3_-H [33]. After analysis of the CCCH gene family in *Arabidopsis* and rice, these CCCH zinc finger motifs were redefined as C-X_4–15_-C-X_4–6_-C-X_3_-H which are glycine-rich and phenylalanine-rich sequences [8]. As shown in this study, *ZmC3H3* contains seven copies of highly conserved CCCH motifs. Moreover, a novel C-X_17_-C-X_6_-C-X_3_-H motif was identified in *ZmC3H17*. To our knowledge, these striking features have not been reported in previous studies. Generally speaking, these features are perhaps exceptions, but they seem to characterize a new CCCH-type zinc finger motif/protein in maize. To confirm this suggestion, further biological experiments should be performed to examine their biological functions in future studies. In maize, 79.4% of all identified CCCH motifs are C-X_7–8_-C-X_5_-C-X_3_-H motifs, and similar results were also found in the *Arabidopsis* and rice CCCH gene families. Among the 68 maize CCCH proteins, 42 members contain the C-X_8_-C-X_5_-C-X_3_-H motif and 37 members contain the C-X_7_-C-X_5_-C-X_3_-H motif, suggesting the crucial functional roles of these motifs in the CCCH proteins. A total of 180 CCCH zinc finger motifs were identified in the maize CCCH family, more than those of *Arabidopsis* (152) and rice (150) [8]. Therefore, about 30 additional motifs were found in the maize CCCH family compared to the *Arabidopsis* and rice CCCH families, despite the three different plants having similar numbers of CCCH proteins. To date, the origin of the 30 additional motifs in maize is unknown, but we can speculate that they are perhaps a result of plant adaptation to various regulatory processes during the evolution of the genome. These novel observations provide important references for exploring the evolutionary history and functional mechanisms of CCCH proteins in plants.

Among the maize CCCH proteins phylogenetically analyzed in this study, it was difficult to assign some genes into groups due to their low bootstrap values in the clades (bootstrap values <50%). In fact, the unclear phylogenetic relationship with low bootstrap support is similar to the phylogenetic analysis of CCCH proteins in *Arabidopsis* and rice. Although sequence analysis revealed that the CCCH motif is a highly conserved functional unit, their other characteristics are highly diverse, especially in the different numbers of CCCH motifs contained in each protein and the spacing in the protein sequences between adjacent CCCH motifs and adjacent cysteines of the zinc finger motif in each sequence. Notably, the majority of CCCH proteins clustered in the same groups displayed a similar domain architecture, suggesting their similar subfamily-specific functions. A combined N-J tree was also constructed to investigate the phylogenetic relationships of CCCH proteins in the three representative species and their evolution-function relationship. We noted that some ortholog proteins displayed a closer relationship than paralog proteins among maize and rice CCCH proteins, suggesting that the ortholog pairs may have originated from a common ancestor when duplication events occurred before the divergence of the grasses. Moreover, monocot (maize and rice) and dicot (*Arabidopsis*) ortholog pairs were also found in our phylogenetic analysis, indicating that these ortholog pairs originated from common ancestral genes that existed before the divergence of monocots and dicots. These observations suggested that the ortholog genes are highly conserved during the evolutionary process. Some groups of CCCH genes were further expanded across the three species. We concluded that the expanded CCCH genes may imply a correlation between gene number expansion and their important roles in plant growth and development.

Wang et al. (2008) previously showed that segmental duplication was largely responsible for the expansion of rice and *Arabidopsis* CCCH gene families [8]. In our study, a total of 15 sister gene pairs of maize CCCH proteins were determined to be involved in segmental duplications by shared phylogenetic clade combinations within the same groups and by locations within the segmental duplicated blocks [22]. No obviously clustered CCCH genes were detected on the 10 chromosomes besides the two sister pairs *ZmC4H13*/*14* and *ZmC4H46*/*47*. These results suggest that segmental duplication is the main contributor to the expansion of the maize CCCH family, which is consistent with the analysis of the *Arabidopsis* and rice CCCH families [8]. It is believed that segmental duplication events occur more often in the more slowly evolving gene families during the process of evolution [34]. The prevalence of ortholog pairs and segmental duplication in the three different species suggested that the CCCH gene family is a conserved and slowly evolving family in plant genomes. This suggestion also gives us a possible reason or explanation for the very similar number of CCCH proteins in the three species, although the genome size of maize is approximately 18 and 6 times as that of *Arabidopsis* and rice, respectively [19], [35]. Indeed, gene duplication, including segmental and tandem duplications, is one of the primary driving forces throughout the evolutionary process of genomes [36]. Previous studies showed that the maize genome has undergone several rounds of whole genome duplication, including an ancient duplication prior to the divergence of grasses (∼50–70 Mya) and an additional whole genome duplication (∼5 Mya), which distinguishes maize from sorghum [37], [38]. By calculating the duplication dates of duplicated gene pairs, we demonstrated that all of the segmental duplication events in the maize CCCH family occurred after the divergence of the grasses.

Plant growth and productivity are frequently threatened by environmental stresses such as drought, high salinity and low temperature during their life cycles. Many stress-related genes are induced in order to adapt to these environmental stresses [39], [40]. In this study, seven maize CCCH genes were predicted to be stress-related genes based on phylogenetic and sequence analysis, and their expression patterns were investigated in five representative tissues and under ABA and drought treatments. The results showed that the seven maize CCCH genes were found to be broadly expressed in almost all tissues examined. Although the seven genes have close evolutionary relationships, they showed largely differential expression patterns in the five tissues. This finding suggested that these genes may function at different stages of plant growth and development. In addition, all seven genes were found to be responsive to ABA treatment or drought stress, and *ZmC3H54* was strongly inducted by both stimuli. We concluded that *ZmC3H54* may play an essential role in response to abiotic stresses. This conclusion was supported by the close relationship of *ZmC3H54* to ATSZF1, ATSZF2 and CaKR1, which were previously identified as stress-response genes [16], [29]. We noted that the two segmental duplication genes, *ZmC3H4* and -*28*, exhibited similarly expression levels in response to ABA treatment. Although the peak expression levels of *ZmC3H4* and -*28* were found at different time points, they were largely up-regulated under drought treatment. Meanwhile, the Ka/Ks ratio of this segmental pair was only 0.109. Therefore, we concluded that *ZmC3H4* and -*28* may exert redundant functions in response to abiotic stress. We also noted that some genes were not only induced by the two stress treatments, but they were also abundantly expressed in specific tissues. These results suggested that these genes may be important both for stress responses and developmental processes. Intriguingly, the results of qRT-PCR were not always consistent with those of the promoter sequence analysis. For example, the promoter analysis showed that *ZmC3H4* and -*34* contain one type of stress-responsive *cis*-element in their promoter regions (Fig. 7), but these genes were induced by both ABA and drought treatments. Thus, the possible regulatory mechanisms of *ZmC3H4* and -*34* in responses to stresses remain unclear. We concluded that some unidentified stress-responsive *cis*-elements may be present in the promoters of these two genes. Alternatively, *ZmC3H4* and -*34* may be secondary targets, which are indirectly regulated by ABA or drought. We will experimentally examine their biological functions in future studies.

In conclusion, CCCH-type zinc finger proteins have essential functions in various developmental processes in plants. To date, the regulatory mechanisms of CCCH proteins in plants remain poorly understood. Therefore, the systematic analysis of the CCCH gene family provides an important reference for future studies on the biological functions of maize CCCH proteins.

## Materials and Methods

### Identification and Sequence Analysis of CCCH Proteins in Maize

Our identification of non-redundant maize CCCH zinc finger proteins was performed using the following strategy. Maize genome sequences were downloaded from http://www.maizesequence.org/index.html, and then DNATOOLS software was used to construct a local database from the nucleotide sequences and protein sequences of the latest complete maize genome. Sequences of *Arabidopsis* and rice CCCH proteins representing different CCCH motif types of C-X_4–15_-C-X_4–6_-C-X_3_-H were used as queries to search against the maize protein database with BLASTP program [8]. All hits with E-values below 0.001 were selected for further analysis, and this step was crucial to identifying all possible maize CCCH proteins. All candidate sequences that met the standards were confirmed to be real CCCH proteins by Pfam (PF00642) (http://pfam.sanger.ac.uk/) and SMART (Sm00356) (http://smart.embl-heidelberg.de/) [41], [42]. Finally, all of the confirmed CCCH proteins were aligned using ClustalW [43], and all identical sequences were checked manually to remove redundant sequences.

Information on maize CCCH genes, including BAC number, chromosomal location, coding sequence (CDS), exons and introns number, ORF length and amino acid (AA), was obtained from the maize B73 sequencing database. The molecular weight (kDa) and isoelectric point (PI) of each gene were calculated by online ExPASy programs (http://www.expasy.org/tools/). Sequences logos of the CCCH zinc finger motifs were produced by online WebLogo software [44]. Exon-intron structure analysis was deduced using GSDS (http://gsds.cbi.pku.edu.cn/) [45]. Maize CCCH genes were placed on chromosomes according to their starting positions given in the maize B73 sequencing database. Chromosome location image was generated by MapInspect (http://www.plantbreeding.wur.nl/uk/software_mapinspect.html). To predict *cis*-acting regulatory DNA elements (*cis*-elements) in promoter regions of CCCH genes, the PLACE website (http://www.dna.affrc.go.jp/PLACE/signalscan.html) was adopted to identify putative *cis*-elements in the 2,000 bp genomic DNA sequences upstream of the initiation codon (ATG) [46].

Full-length sequences of maize, rice and *Arabidopsis* CCCH proteins were aligned using ClustalX v1.83 [47]. Phylogenetic trees were conducted using MEGA 4.0 [48]. The neighbor-joining (N-J) method was used to construct different trees with the pairwise deletion option. For statistical reliability, bootstrap analysis was carried out with 1,000 replicates to evaluate the significance of each node. For detection of tandem and segmental duplications, paralogs were regarded as tandem duplicated genes provided two maize CCCH genes were separated by five or fewer gene loci according to the maize B73 genome annotation. Paralogs were designated as segmental duplicated genes if they were placed on duplicated chromosomal blocks as previously proposed by Wei et al. (2007) [22], [49], [50], [51].

The number of nonsynonymous substitutions per nonsynonymous site (Ka) and the number of synonymous substitution per synonymous site of duplicated genes were calculated by DnaSP v5.0 [52]. The ratio of nonsynonymous to synonymous nucleotide substitutions (Ka/Ks) between paralogs was analyzed to detect the mode of selection. To estimate the times of duplication events, the Ks value was translated into duplication time in million years based on a rate of λ substitutions per synonymous site per year. The duplication time (T) was calculated as T  =  Ks/2λ×10^−6^ Mya (λ = 6.5×10^−9^ for grasses) [23], [24].

### Plant Materials, Growth Condition and Stress Treatments

The maize inbred line B73 was used to check the gene expression levels in all experiments. Five representative tissues (10-day-old root, 3-week-old leaf, 6-week-old stem, 5–8-cm young ear and 6–10-cm silk) were collected from a life cycle of maize. For expression analysis of maize CCCH genes under stress, plants were grown in a greenhouse with a 14-h light/10-h dark cycle at 28–30°C. The drought treatment was performed following the method described in our previous study [51]. For ABA treatment, 3-week-old seeding leaves were sprayed with 100 µM ABA solution and sampled at 0, 3, 6 and 12 h after treatment [53], [54]. For all the stages, three biological replicates were conducted for each sample.

### RNA Extraction and qRT-PCR Analysis

Total RNAs of all the collected samples were extracted using the Trizol reagent (Invitrogen) according to the manufacturer’s instructions. The DNase-treated RNA was reverse-transcribed using M-MLV reverse transcriptase (Invitrogen). qRT-PCR was performed on an ABI 7300 Real-Time system (Applied Biosystems). The gene-specific primers designed using Primer Express 3.0 software (Applied Biosystems) were employed to amplify 90–150 bp PCR products unique to each gene (Table S3). Each reaction contained 12.5 µl of 2×SYBR Green Master Mix reagent (Applied Biosystems), 2.0 µl of diluted cDNA sample, and 400 nM gene-specific primers in a final volume of 25 µl. The thermal cycle was used as follows: 95°C for 10 min, followed by 40 cycles at 95°C for 15 s and 60°C for 1 min. After the PCR was completed, a melting curve was generated to analyze the specificity for each gene by increasing the temperatures from 60 to 95°C. Three technical replicates were performed for each gene. Expression level of the maize *Actin 1* gene was used as an internal control. The relative expression level was calculated as 2^–ΔΔ*C*T^ [Δ*C*_T_  =  *C*_T, Target_ – *C*_T, *Actin 1*_. ΔΔ*C*_T_  =  Δ*C*_T, treatment_ – Δ*C*_T, CK (0h)_]. The relative expression level (2^–ΔΔ*C*T, CK (0h)^) in the normal plant without stress treatment was normalized to 1 as described previously [55]. Statistical analyses were performed using SDS software 1.3.1 (Applied Biosystems).

## Supporting Information

Table S1 **CCCH gene family in maize.**
(DOC)

Table S2 **Detailed information of CCCH proteins in maize.**
(XLS)

Table S3 **Maize CCCH gene-specific primers used for qRT-PCR analysis.**
(XLS)
