# Supplementary material for: CCCH-Type Zinc Finger Family in Maize: Genome-Wide Identification, Classification and Expression Profiling under Abscisic Acid and Drought Treatments
Source: PLoS One. 2012 Jul 6;7(7):e40120. doi: 10.1371/journal.pone.0040120 (PMC3391233; doi:10.1371/journal.pone.0040120)
Supplement: Table S1 — CCCH gene family in maize. (DOC) [file pone.0040120.s001.doc]

**Table S1.** CCCH gene family in maize

| **Gene**  **Name^a^** | **BAC No.** | **ORF**  **(bp)^b^** | **Number of**  **CCCH motifs** | **Deduced polypeptide^c^** | | | **Chr.^d^** |
| --- | --- | --- | --- | --- | --- | --- | --- |
|  |  |  |  | **Length**  **(aa)** | **MW**  **(kDa)** | **PI** |  |
| ZmC3H1  ZmC3H2  ZmC3H3  ZmC3H4  ZmC3H5  ZmC3H6  ZmC3H7  ZmC3H8  ZmC3H9  ZmC3H10  ZmC3H11  ZmC3H12  ZmC3H13  ZmC3H14  ZmC3H15  ZmC3H16  ZmC3H17  ZmC3H18  ZmC3H19  ZmC3H20  ZmC3H21  ZmC3H22  ZmC3H23  ZmC3H24  ZmC3H25  ZmC3H26  ZmC3H27  ZmC3H28  ZmC3H29  ZmC3H30  ZmC3H31  ZmC3H32  ZmC3H33  ZmC3H34  ZmC3H35  ZmC3H36  ZmC3H37  ZmC3H38  ZmC3H39  ZmC3H40  ZmC3H41  ZmC3H42  ZmC3H43  ZmC3H44  ZmC3H45  ZmC3H46  ZmC3H47  ZmC3H48  ZmC3H49  ZmC3H50  ZmC3H51  ZmC3H52  ZmC3H53  ZmC3H54  ZmC3H55  ZmC3H56  ZmC3H57  ZmC3H58  ZmC3H59  ZmC3H60  ZmC3H61  ZmC3H62  ZmC3H63  ZmC3H64  ZmC3H65  ZmC3H66  ZmC3H67  ZmC3H68 | AC194575.2  AC209839.3  AC199953.4  AC206947.3  AC211892.4  AC206638.3  AC203215.3  AC225226.3  AC195237.3  AC202954.4  AC209023.3  AC214002.3  AC201805.4  AC194118.3  AC194171.3  AC217295.3  AC196780.4  AC212898.4  AC187829.3  AC196257.3  AC204644.3  AC191719.3  AC191273.3  AC211198.5  AC191337.4  AC204231.3  AC196154.3  AC231546.3  AC197023.2  AC231943.3  AC197550.3  AC230010.2  AC212191.4  AC233871.1  AC204923.4  AC195867.2  AC194411.3  AC234575.4  AC202882.3  AC205072.3  AC202899.3  AC216049.3  AC225308.2  AC187872.3  AC194344.5  AC198588.3  AC198588.3  AC211875.4  AC193477.3  AC190806.4  AC195174.2  AC202538.3  AC210815.5  AC206300.3  AC199860.4  AC216825.1  AC195828.3  AC211407.4  AC210292.3  AC211252.4  AC211745.5  AC200063.3  AC214431.3  AC216071.3  AC188829.3  AC205828.4  AC203750.3  AC200297.4 | 1194  2028  2226  2241  897  924  1008  2562  1332  1083  900  1113  1356  1416  519  1293  1098  1056  2076  933  1893  1332  648  2094  996  1077  1362  1449  741  1182  1206  819  1284  1251  1185  756  906  1185  813  924  957  864  1971  1320  1986  1416  1347  3024  501  774  1137  915  789  1119  1002  1089  1284  1110  3579  1023  2055  1635  1785  1092  2889  987  921  2040 | 2  1  7  2  2  3  6  1  5  2  2  2  5  5  1  5  2  2  3  4  1  5  3  1  1  3  1  2  3  1  1  1  2  2  1  3  3  2  1  2  2  2  2  1  1  5  5  2  1  2  2  2  1  1  5  2  5  4  5  3  1  5  2  3  3  6  3  3 | 397  675  741  746  298  307  335  853  443  360  299  370  451  471  172  430  365  351  691  310  630  443  215  697  331  358  453  482  246  393  401  272  427  416  394  251  301  394  270  307  318  287  656  439  661  471  448  1007  166  257  378  304  262  372  333  362  427  369  1192  340  684  544  594  363  962  328  306  679 | 42.67  71.66  83.44  79.77  34.48  31.89  37.51  92.59  48.04  37.92  34.54  39.79  48.44  49.75  19.73  46.83  41.67  38.11  74.17  34.20  67.69  47.83  23.29  74.69  36.13  37.33  52.22  52.09  26.81  45.05  44.03  30.42  46.03  45.44  45.38  27.29  31.25  42.10  29.69  35.57  34.41  33.67  69.82  50.29  70.40  49.73  48.62  112.96  18.00  27.94  40.18  35.28  28.03  40.03  36.76  39.42  46.35  41.77  131.13  36.29  72.22  58.78  62.66  38.37  107.00  36.85  31.78  76.68 | 6.42  6.03  5.19  6.40  9.00  9.52  9.87  5.47  8.92  6.37  9.23  6.46  8.06  6.27  4.76  8.76  5.28  8.64  5.18  8.47  5.82  8.78  9.12  5.87  7.90  8.03  8.76  7.97  8.53  8.11  8.81  5.41  8.13  8.78  9.56  8.33  9.51  8.02  5.67  9.22  7.11  9.22  6.88  6.68  6.84  7.22  7.84  6.41  8.61  8.58  6.46  9.01  6.40  8.65  8.24  10.44  8.68  8.12  9.01  9.11  6.21  6.66  5.71  7.14  9.44  9.47  9.43  5.33 | 1  1  1  1  1  2  2  2  2  2  2  3  3  3  3  3  3  3  3  3  3  4  4  4  4  4  4  5  5  5  5  5  5  6  6  6  6  6  6  6  6  7  7  7  7  8  8  8  8  8  8  8  8  8  8  8  8  9  9  9  9  10  10  10  10  10  10  10 |

^a^ Systematic nomenclature of maize CCCH genes.

^b^ Length of open reading frame in base pairs.

^c^ Length (number of amino acids), molecular weight (kilodaltons), and isoelectric point (pI) of the deduced polypeptides.

^d^ Chromosomal localization of maize CCCH genes.
